# Training Top-Down Attention Improves Performance on a Triple-Conjunction Search Task

**DOI:** 10.1371/journal.pone.0009127

**Published:** 2010-02-18

**Authors:** Farhan Baluch, Laurent Itti

**Affiliations:** 1 Neuroscience Graduate Program, University of Southern California, Los Angeles, California, United States of America; 2 Department of Computer Science, University of Southern California, Los Angeles, California, United States of America; Ecole Polytechnique Federale de Lausanne, Switzerland

## Abstract

Training has been shown to improve perceptual performance on limited sets of stimuli. However, whether training can generally improve top-down biasing of visual search in a target-nonspecific manner remains unknown. We trained subjects over ten days on a visual search task, challenging them with a novel target (top-down goal) on every trial, while bottom-up uncertainty (distribution of distractors) remained constant. We analyzed the changes in saccade statistics and visual behavior over the course of training by recording eye movements as subjects performed the task. Subjects became experts at this task, with twofold increased performance, decreased fixation duration, and stronger tendency to guide gaze toward items with color and spatial frequency (but not necessarily orientation) that resembled the target, suggesting improved general top-down biasing of search.

## Introduction

Bottom-up, stimulus-driven processes as well as top-down, goal-driven processes exert influence on perception and therefore on the ability to perform visual tasks. Experts in a wide range of fields [1], from radiologists detecting tumors [2], image analysts screening baggage at the airport [3], pilots scanning their instrument panel [4], to chess grand masters [5] rely on their perceptual discrimination and selection abilities to make judgements often in life threatening situations. Tasks performed by these experts rely on both bottom-up and top-down processes to search for and direct attention towards features of the image that are crucial to enabling perceptual judgement with confidence. The central question in this study is whether, and to what extent, training and expertise improve, or otherwise modify, how rapid top-down goal-driven tuning of visual processing can enhance visual information for perceptual decisions, specially in feature rich enviornments.

Guidance of visual search for features in an image by top-down processes poses a constant demand on the visual and attentional systems to convert descriptions of desired target(s), which may change from moment to moment depending on behavioral goals, into appropriate guiding signals that can facilitate localization of a target. The quality of the guidance is determined by a number of factors including, i) the properties of the tuning functions of the sensory system [6], ii) the ability of the sensory system to eliminate noise [7], and iii) the discriminability of the target from distractors and background clutter (signal-to-noise ratio). On a short time scale, attention can enhance guidance through enhanced gain [8], enhanced spatial resolution [9], effective stimulus strength [10], or noise exclusion [7]. Analogous effects have been observed in perceptual learning studies over a longer time scale of up to a few days or longer.

Perceptual learning studies have shown that practice can improve performance in discrimination [11]–[14] and detection [15], [16]. These studies have shown improvement in either a spatially or featurally specific manner and thus implicated early sensory cortex as the locus of plasticity and this has also been observed in electrophysiological studies [17], [18]. Although most studies limit their training to either specific spatial locations or specific stimulus feature ranges, there has been some speculation about mechanisms of more general improvement in tasks. Some studies for example, have implicated the higher cortex [19]–[21] in learning. Plasticity effects have been observed in later visual areas, namely V4 and FEF (frontal eye fields), as a result of perceptual learning [22], [23]. Learning in tasks such as visual search has also been shown to be less specific [24]. Sireteanu et al. [25] have shown non-specificity of perceptual learning effects specially in visual search tasks, and thus placed the locus of plasticity for learning a visual search task at a higher level than sensory cortices. One question which has remained outstanding, however, is whether training can improve the effectiveness of the dynamic top-down attention biasing process itself through what has been termed process-based learning [26], as opposed to exhibiting sharper visual discrimination abilities for a specific type of target or location (perceptual learning or automaticity through better memory retrieval [26]), or generally improving speed and/or performance on a task (task acquisition for search). This type of non-specific learning remains understudied and more specifically, the pairing of learning within a visual search task to observe the effects of training top-down attention remains relativity unexplored (although see [27]).

In this study we address the question of whether expertise can be gained in a triple-conjunction (color, spatial frequency, and orientation) search task when both the features and spatial location of the target are changed from trial to trial while maintaining a persistent level of bottom up uncertainty in the Shanon entropy sense. This imposes a novel and interesting new constraint on the type of learning that can occur, eliminating the cases of (perceptual) learning due to ‘stimulus imprinting’ [28] and focusing on what Goldstone [28] has termed ‘attention weighting’. Specifically, this type of paradigm makes a demand on the observers to make fast trial-by-trial adjustments of top-down biasing weights in order to succeed in the search task. We also ask what difference, if any, training makes on the subjects’ saccadic eye movements and the types of distractors that they look at. This is a departure from a typical learning paradigm where the stimulus set is often restricted in either space or feature set. We look for mechanisms of acquisition of general domain expertise when the observers are given a task that requires attention to the stimulus in order to achieve success. By analyzing eye movements we can ensure that effects beyond general task acquisition are captured. Changing the target on each trial puts the spotlight on mechanisms of attentional biasing efficacy rather than simple perceptual learning. We hypothesized that better biasing would lead to increased guidance towards items that are similar to the target as the biasing process would render items sharing features with the target more salient. Thus the number of items that were viewed need not necessarily be reduced but the quality of the set may improve. An alternate outcome would be that subjects view a smaller number of items which would suggest a trend toward automaticity or more pre-attentive guidance.

We show that learning occurs even when the target is changed in both features and spatial location on every trial. The improvement is marked by a decrease both in intersaccadic interval (ISI) and reaction time. The decrease in ISI suggests an improvement in discrimination and a stronger emphasis on the selection (detection) task. However, we did not observe a significant drop in saccade counts which suggests that the improvement in selection was limited to improving the ‘quality’ of the subset of items on the display that are scrutinized (the size of the subset remaining fairly consistent). We also find that subjects tend to exploit two of the three features of the stimuli, making saccades towards items that are similar to the target in color and spatial frequency but, interestingly, not necessarily in orientation.

In sum, our results provide evidence for a mechanism of expertise acquisition that is driven by production of better top-down biasing signals, the behavioral correlate of which is the increased similarity effect observed. This coupled with improved discrimination, likely driven by multiple exposures to the family of stimuli used in the task, define the enabling mechanisms that allow the transition from novice to expert.

## Methods

### Ethics Statement

Subjects gave written consent under a protocol approved by the Institutional Review Board of the University of Southern California, and were paid for participating in the study.

### Subjects

Human subjects recruited for this study were undergraduate and graduate students at University of Southern California. Subjects included four males and one female aged 21–26 years. All subjects had normal or corrected vision. Subjects gave written consent under a protocol approved by the Institutional Review Board of the University of Southern California, and were paid for participating in the study. Subjects were naive to the purpose of the experiment and had never seen any of the stimuli before.

### Stimuli

**Figure 1:**
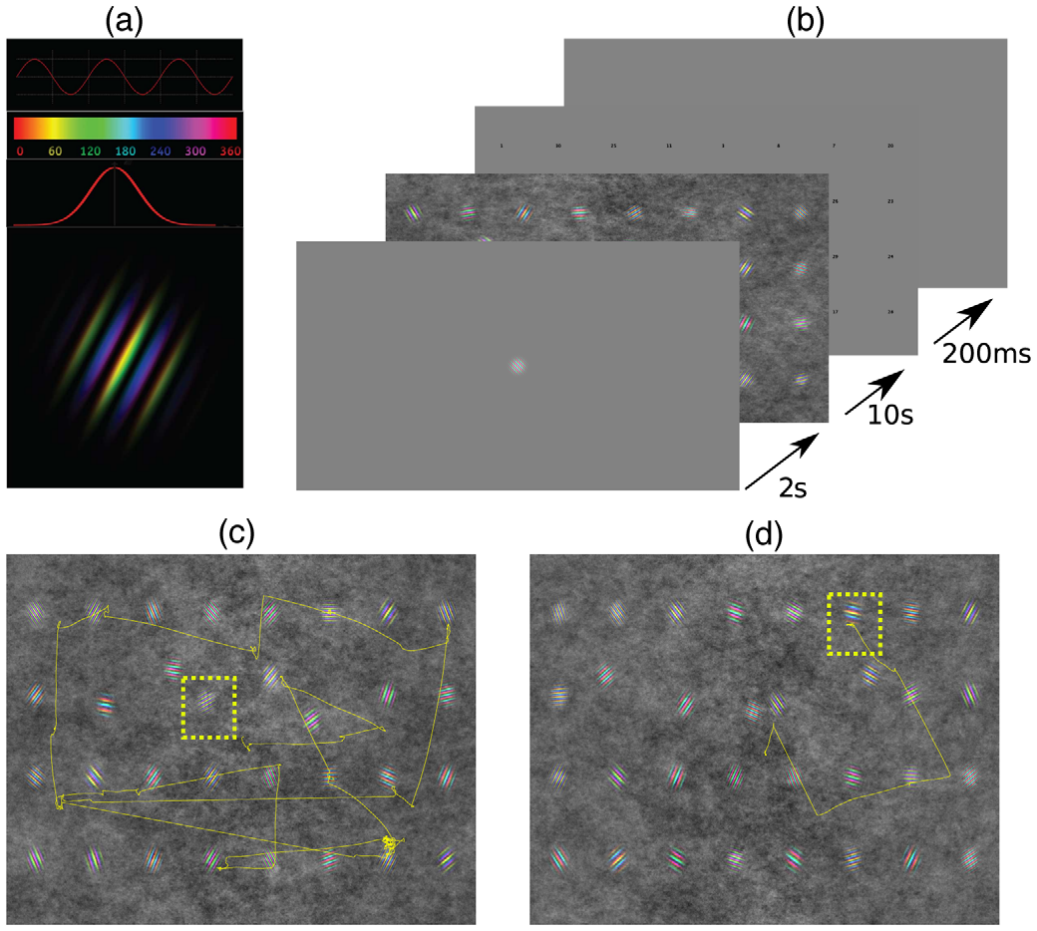
Stimulus and Paradigm. (a) Color Gabor patches constructed by first applying a gaussian envelope over a sinusoid as shown. At each point the phase of the sinusoid was used to modulate a hue axis in the HSV color space. (b) A trial started with a two-second target preview followed by a display of the search array for a maximum of ten seconds. If subjects found the target before the 10 seconds elapsed they hit a key to move to the next display. The next display showed numbers corresponding to Gabor patch locations in the search display. The numbers were displayed for only 200ms to ensure that subjects fixate the target in order to report the correct number. Subjects were then aske d to report the number at the target location. (c) A typical eye trace overlayed on a search array, showing an early trial. (d) A typical eye trace overlayed on a search array, showing a late trial.

A set of colored Gabor patches were designed for this experiment, which provided the ability to vary features along three dimensions: color, spatial frequency, and orientation. The luminance profile of each Gabor patch is given by the following equation:
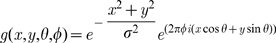
(1)where 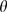 is the orientation of the patch, 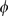 is the spatial frequency. Each patch subtended 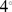 of visual angle. The phase of the sinusoid at each point was used to modulate the color of the pixels along the hue axis in the HSV color space, as shown in figure 1a. By sliding a window along the hue axis, the range of colors in the patch was changed, thus modifying the appearance of the patch. The window spanned from 0 to 360 and a hue shift essentially recentered the window around a given value. Each Gabor patch was then defined by its spatial frequency which ranged from 1.7 c/deg to 5.2 c/deg, orientation, which ranged from 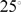 to 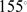, and finally a color hue value that determined the shift of the hue window.

Search arrays were constructed from 32 Gabor patches embedded in 1/f noise in a 4×8 grid, with slight spatial jitter (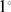 along the x or y direction) applied to each patch. One of the Gabor patches was randomly chosen as the target for each search array.

### Paradigm

Subjects conducted 1,000 trials of visual search over the course of ten consecutive days. Each day consisted of a session of 100 trials with a break after 50 trials. Stimuli were presented on a large (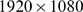 pixels) LCD monitor (Sony Bravia XBR-III) and subjects were seated in a comfortable chair with their head stabilized by a chin rest. The viewing distance was 97.8 cm, corresponding to a field of view of 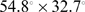. A typical trial, as illustrated in figure 1b, began with a fixation cross at the center of the display followed by a 2 second target preview, presented at the center with a gray background. The gray value of this background was equal to the mean gray of the 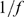 noise of the corresponding search array display. Subjects were instructed to find the target as fast and accurately as possible and had a maximum of ten seconds to find the target. Their eye movements were recorded as they searched for the target (see below for eye-tracking methods). Upon locating the target, subjects pressed a response button, at which point the search array disappeared. A display consisting of numbers that corresponded to the Gabor patch locations was then displayed for 200ms. Subjects had to read and key-in the number at the location of the target using a keyboard. The font size was sufficiently small that one could not read the numbers corresponding to one Gabor patch while fixating at the location of any other Gabor patch. The goal of this ‘no cheat’ procedure was to ensure that subjects reported correctly the patch which they thought was the target(for more details on this procedure see [29]). After subjects provided input, they were given feedback as a ‘correct’ or ‘incorrect’ response, as well as the current level of performance (% correct responses so far). Each session lasted approximately 45 minutes.

### Stimulus Presentation and Eye-Tracking Procedures

The subjects’ eye movements were recorded as they searched for the target in the search array. Eye movements were recorded at a sampling frequency of 240 Hz, using an infrared-video-based eye-tracker (ISCAN RK-464) and the pupil and corneal reflection from the right eye were used to determine the gaze position with an accuracy of 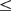 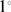. Calibration was performed using an online system that presented subjects with a central fixation point followed by a point at one of nine locations on a 3×3 grid. Subjects had to saccade from the central fixation point to one of the nine locations and maintain stable fixation (x and y position variance 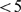 pixels) for 300ms (75 samples). Once stable fixation was established the next location was presented. This process was repeated until stable fixations at all nine points were found. The eye positions obtained were then used to perform an affine transform and the transformed eye positions were displayed on the screen for the experimenter to confirm that an accurate calibration session had been conducted. During offline analysis a further thin-plate-spline interpolation [30] was performed to obtain accurate transformation from eye-tracker coordinates to screen coordinates. A recalibration session was performed every 20 trials to correct for possible head movements. Once transformed, the eye-traces could be overlaid on the images for further analysis as shown in figure 1(d).

### Data Analysis

The subjects’ eye movements were calibrated as described above and an algorithm was used to parse the eye movements into saccades using a combination of filtered instantenous velocity measurements and a simple windowed Principal Components Analysis (PCA). Eye movement segments with a minimum velocity 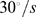 and a minimum amplitude of 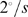 were classified as saccades. Blinks were identified by a pupil diameter reading of zero and trials with either blinks or loss of tracking for more than 10% of the trial were removed from further analysis. Unfortunately, on day two, one of the subjects’ eye movements were lost due to machine failure; however, he completed all trials and continued to participate in the study. This loss not withstanding, we retained 97% of the 4,900 available trials, obtaining a total of 76,287 saccades for analysis.

We performed analysis on changes over time in the subjects’ eye movements by constructing feature similarity maps and correlating these with binary saccade maps. The feature similarity maps were constructed as follows. We first discretized the feature space by dividing each dimension into ten bins (several numbers were tried for this and numbers between 10–25 bins gave similar results). Each Gabor patch was then defined as a triplet of bin values 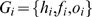 where 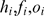 are the bins of hue, frequency, and orientation respectively of Gabor patch 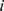. A feature similarity map for each trial consists of 32 cells arranged in a 4×8 grid each corresponding to one of the color Gabor patch in the search array for that trial. Similarity maps for each feature were constructed individually. A feature similarity map for hue, for example would contain in each cell 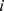 the difference between the hue bin value 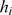 of the Gabor patch and the hue bin 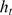 of the target Gabor patch 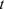 for the trial. In order to maintain an intuitive sense of the similarity measure (high values for high similarity) we computed similarity between the target patch 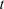 and a Gabor patch 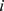 for each feature 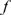 as 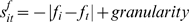 (where granularity was set to ten since we divided the feature space into ten bins). Large values in cells therefore mean that the particular distractor was very similar to the target and vice versa.

As described before we drew the features of the distractors in each display from a uniform distribution and therefore by design the bottom up uncertainty in each display averaged across sessions should remain constant. In order to ensure that this was the case we computed the Shannon entropy in each feature similarity map. This enabled us to quantify the amount of uncertainty in our arrays. We then computed the average entropy per session and ran a regression to look for any trends over time. As expected we found no significant trends (color 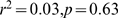; frequency 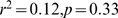, orientation 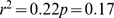).

To construct binary saccade maps we first assigned saccade end points to Gabor patches if the distance from the end point to the center of the Gabor patch was smaller than 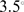. These assignments allowed us to fill a 4×8 grid of cells corresponding to the 4×8 grid of Gabor patches, with 1 for a saccade end point landing on the Gabor patch and a 0 for no saccade towards the patch. In this manner binary saccade maps were constructed and later correlated with the feature similarity maps. When a particular patch was fixated several times we still placed a one in the map in order to retain the binary nature of the saccade maps.

## Results

### Performance

**Figure 2:**
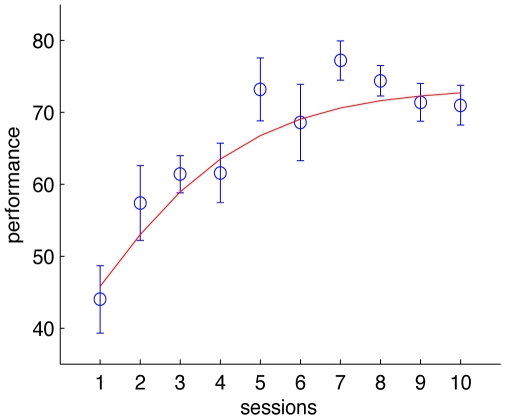
Performance results. Mean percentage correct performance obtained by taking a mean across subjects for each of the 10 sessions. Error bars are SEM across subjects. Smooth curve is a fit to a logistic function (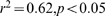).

Measuring performance as the percentage of correct trials for each 100-trial session, we found that subjects showed improved performance over the course of the trials (figure 2). The mean percentage performance of the group was computed by taking an average of the percentage correct responses by each of the five subjects for each session. A one-way ANOVA showed an effect of session on mean performance (F(9,40) = 6.88 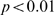). The change in performance measured by the slope (indicative of learning rate) of the logistic fit on the data halfs at day five and later levels off, hovering around 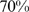 to 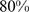 correct as shown in figure 2.

**Figure 3:**
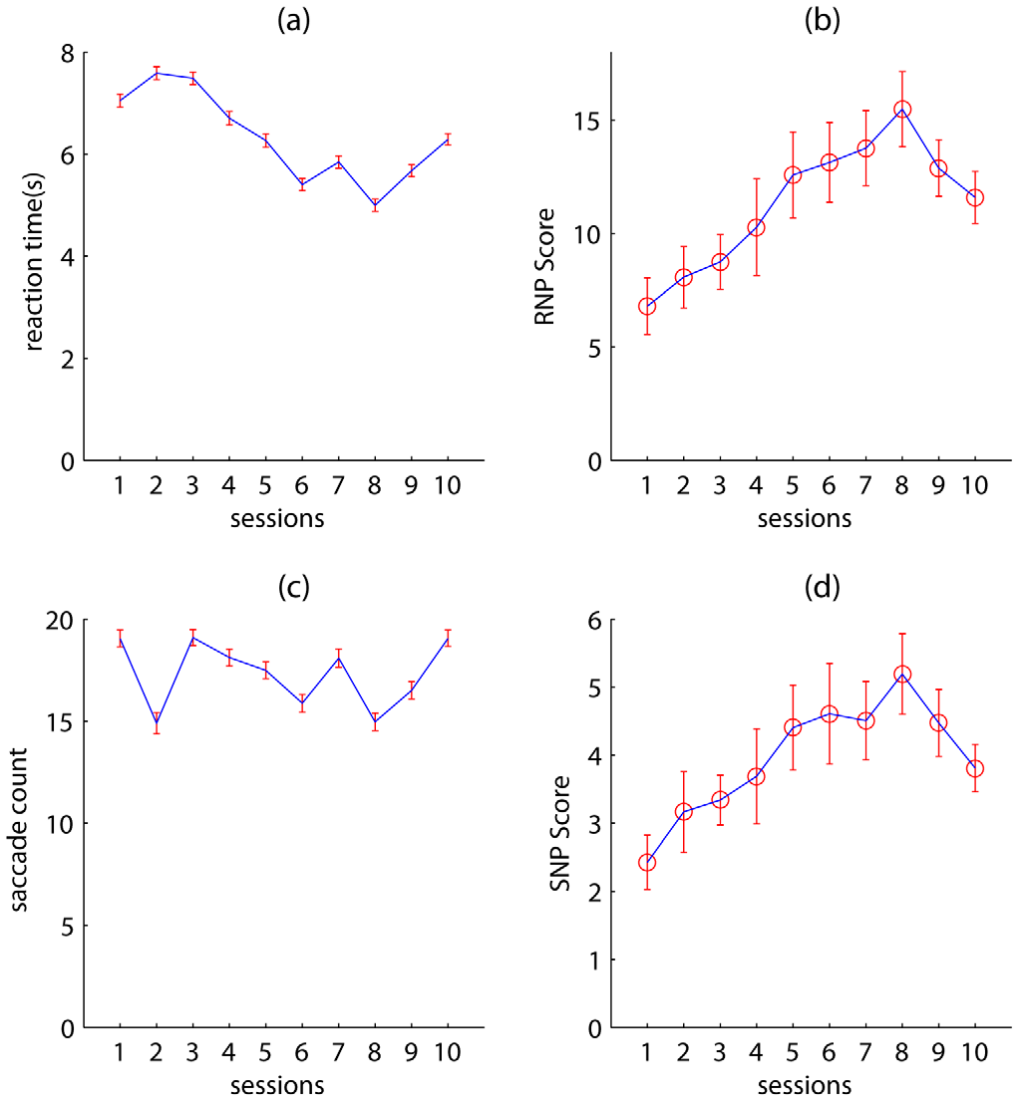
Reaction time and saccade count data. (a) Reaction time plotted as a function of session computed by pooling together all trials by all subjects for each session and taking the mean. Errorbars are SEM. (b) Reaction time Normalized Performance (RNP) score computed by normalizing mean performance by mean reaction time per session. Error bars are SEM taken across sessions. (c) Saccade counts plotted as a function of session, computed by pooling together data from all subjects per session and taking a mean. Errorbars are SEM. (d) Saccade count Normalized Performance (SNP) score computed by normalizing mean performance by mean saccade count per session. Errorbars are SEM.

This indicates that the subjects improved on the task and answered correctly a greater percentage of time after conducting several hundreds of trials of the task, despite the fact that the features and spatial location of the target was changed on every trial. Pooling together the reaction times for each subject and averaging across the sessions revealed an effect of session on the mean reaction time (figure 3a) for our pool of subjects (one-way ANOVA F(9,4990) = 50.71 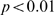). A similar but weaker effect in number of saccades was observed (one-way ANOVA F(9,4766) = 12.62 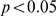) as shown in figure 3c. To ensure that the performance improvements observed were not due to a speed-accuracy tradeoff, we normalized performance by the mean number of saccades and mean reaction time separately. Mean performance normalized by the mean number of saccades gave us a measure of subjects’ per-saccade search efficiency. Plotting this as a function of sessions (figure 3d), we find an increased per saccade efficiency (one way ANOVA F(9,40) = 2.43 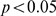). Similarly, plotting mean performance (figure 3b) per session normalized by the mean reaction times we find an upward trend of search performance per unit time spent searching (one-way ANOVA F(9,40) = 3.71 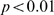). These results show a clear improvement of all subjects on the task with training. To confirm that learning was not just a result of improvement in reporting the numbers in the brief display, we examined the accuracy of reporting the number at the position last fixated. We found that the number at the position of last fixation matched the reported number 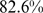 of the time on incorrect trials and 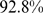 on correct trials. Further pooling the trials together and computing an average over each session, normalized by the number of incorrect trials, we find no effect of session on report accuracy (one-way ANOVA F(9,40) = 0.77, p = 0.65). Thus, we can rule out that performance improvements might have been due to an improved ability to read and report the numbers.

### Differences in Basic Eye Movement Statistics

**Figure 4:**
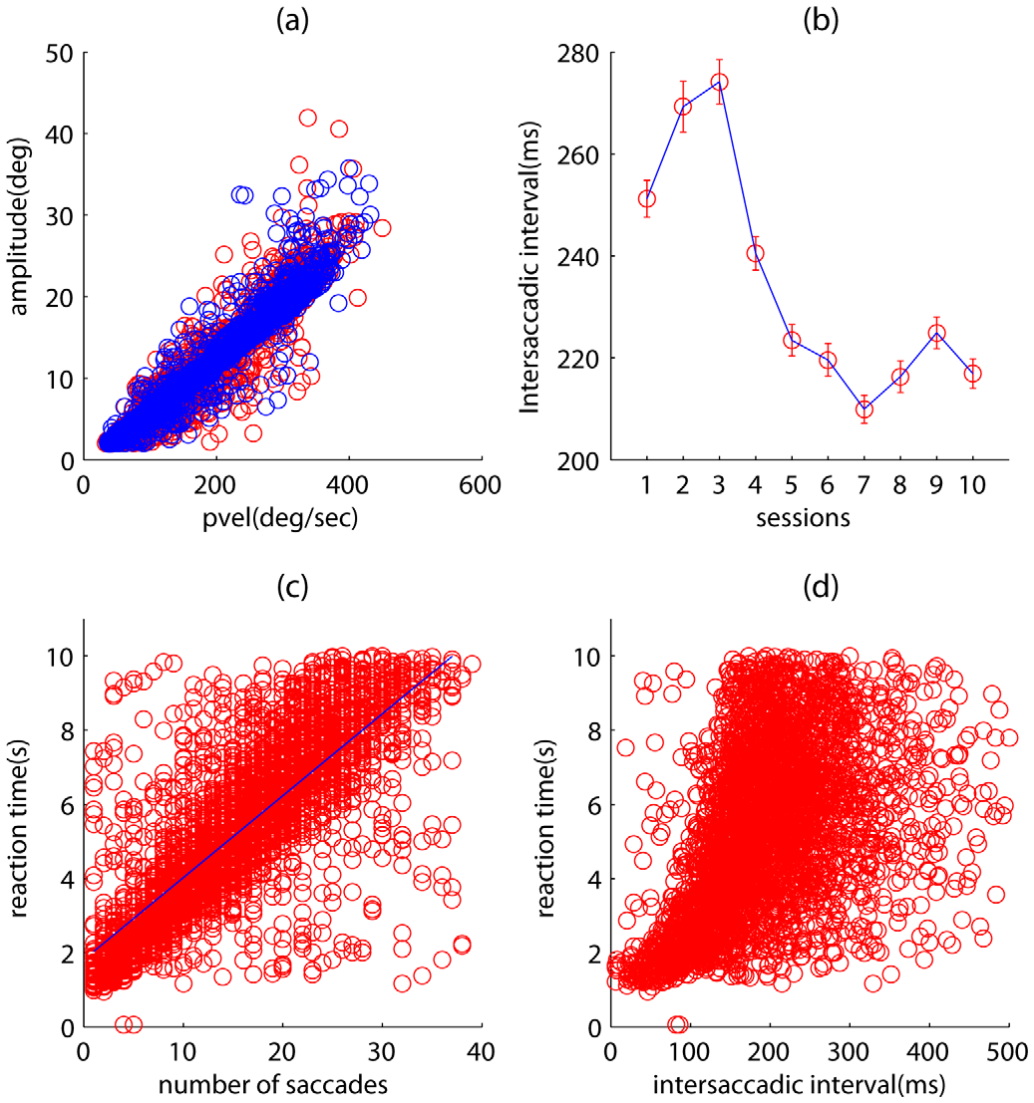
Saccade statistics. (a) Main sequence, plotting saccade amplitudes against peak velocity for the first session (red) and fifth session (blue). Overlap shows no difference in main sequence. (b) Intersaccadic interval reduces with session data. Points were computed by pooling saccades for each session for all subjects and taking a mean. Error bars are SEM. (c) Reaction time as a function of number of saccades. Regression line shows significant correlation (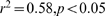). (d) Reaction time as a function of intersaccadic interval. Regression shows weak correlation (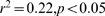).

The eye movements of all the subjects were grouped by session, and statistics were then computed on this data. We first analyzed the main sequence, which plots peak velocity against saccadic amplitude. The main sequences for session one and session five are shown in figure 4a. To determine whether there was a difference between the two sequences we first fitted a linear function to the main sequence of session one and then used this model to predict saccade amplitudes using the peak velocity data from session five saccades. We then ran a two-sample t-test between predicted saccade amplitudes and real saccade amplitudes for session five and found no significant difference (p = 0.50). The analysis of the main sequences therefore revealed no effect of training on these saccade statistics, and the subjects’ eye movements were similar in this regard. Similarly, no significant trend was found in saccadic amplitude or velocity individually (data not shown). However, when we analyzed the ISI we found a significant drop from early sessions in training to late sessions, as illustrated in figure 4b. Specifically, a one-way ANOVA showed a strong effect (F(9,73481) = 43.95, 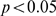) of session on intersaccadic interval. These results demonstrate a change in saccadic strategy on the part of the observes, a change marked by increased efficiency in examining the Gabor patches and greater speed in rejecting non-target Gabor patches. As expected a fall in ISI resulted in a drop in reaction time (RT). However, we found that RT was more strongly dependent on the number of saccades made rather than on ISI. We found a significant dependence (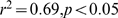) of RT on the number of saccades made (figure 4c). A weaker dependence (figure 4d) of RT on ISI was found (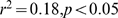). The data shown in the figures is for trials where reaction time was 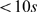; the results for the full dataset were similar (RT vs saccade count 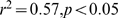 and RT vs ISI 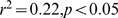). Therefore number of saccades appeared to be more important in determining RT than ISI.

### Individual Feature Similarity Map and Saccade Map Correlations

**Figure 5:**
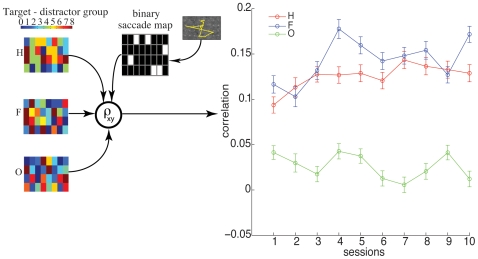
Single feature correlations. Feature similarity maps are shown on the left with hot colors showing high similarity. These similarity maps are correlated with saccade maps to yield a correlation value 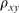. The plot shows mean correlations per session for each feature. Error bars are SEM.

Having constructed feature similarity maps and binary saccade maps, a correlation value between the binary saccade map and each of the feature correlations maps were computed for each trial. Correlation values for each session were computed by pooling together trials of all subjects within a session and then computing the mean. Figure 5 shows that, i) feature similarity maps and binary saccade maps are correlated, and ii) hue and frequency similarity maps become increasingly correlated as the sessions progress, however, no such trend can be observed for orientation. The positive trend indicates correlations between non-zero values in the binary saccade map with high values in the feature similarity maps. This demonstrates a higher likelihood of subjects making saccades towards items that are similar to the target.

The significant increase in correlation of the hue map from session one to session five (paired t-test 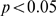) demonstrates that subjects increasingly looked at items that were closer in hue to the target. There was also a significant increase in frequency correlation from session one to session five (paired t-test 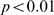), once again demonstrating a tendency to saccade towards items with frequency more similar to the target. This was not the case for orientation, where we found a non-significant (p = 0.36) difference between session one and session five.

**Figure 6:**
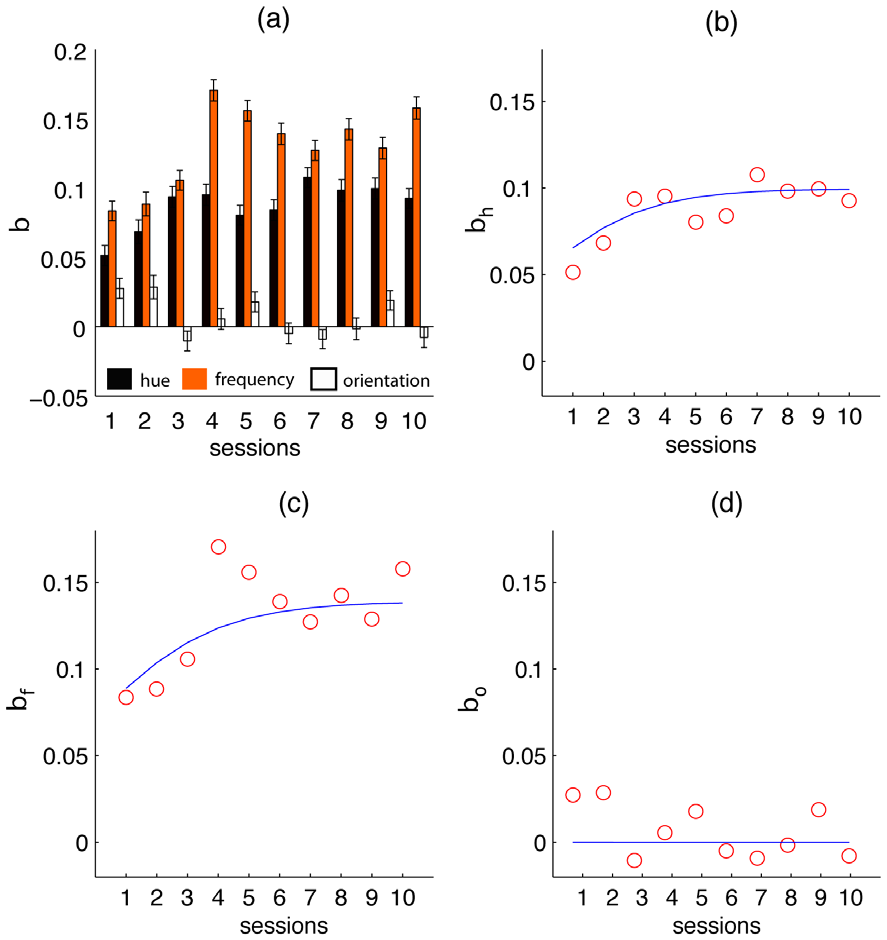
Multiple Logistic regression results. (a) Coeffecient values for each feature plotted as a function of session. (b) Regression line fitted to the coefficient values for hue (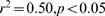), (c) frequency (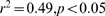) and, (d) orientation (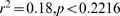).

We further quantified this result by running a multiple logistic regression on the data, examining the combined effect of feature distances on the probability of making a saccade towards the target in a given session. Coefficients obtained from this regression were then plotted as a function of session and fitted to a logistic function 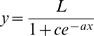 (figures 6a, b, and c), where 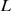 is the upper limit of the curve, and 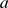 determines the slope of the curve, while 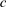 determines shift of the inflection point of the function. 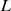 is evaluated by computing an average of the coefficient values for sessions seven through ten. The coefficients’ trends plateau at seven coinciding with a plateau in performance thus we use the mean to compute 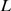. We then linearized the function to run a linear regression that provided a method for computing the parameters 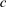 and 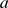. The regressions yielded significant trends for hue (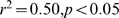), and frequency 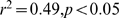 coefficients but not for orientation (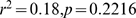).

These results demonstrate a tendency of subjects to exploit hue and frequency as the primary features while giving lowest priority to orientation. This effect has also been observed in previous studies [31]–[33] that found a hierarchy of feature efficacy in biasing saccades towards targets, with color being the dominant feature followed by size and orientation.

### Feature Combination Rules

**Figure 7:**
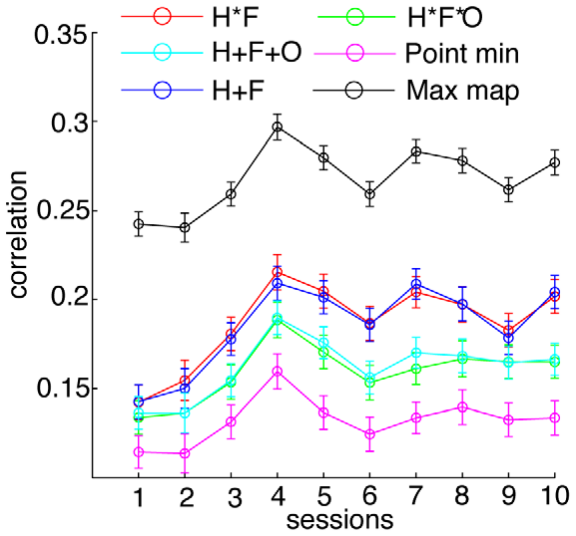
Feature combination correlations. Plots showing correlations of feature similarity maps combined using various methods, as a function of sessions. The black curve (Max map) represents an upper bound computed by taking the most correlated feature map on each trial and computing averages across all trials for each session. The correlation values for this upper bound can be used to compare mean correlation values for all other combination rules H*F (red), H*F*O (green), H+F (blue), H+F+O (cyan) and, point wise minimum rule (magenta).

We also investigated the question of what combinations of features might be learned. Several feature combination rules were tested by combining the similarity maps using different computations. Figure 7 plots the correlation values across the sessions for maps constructed using various methods of combining the individual feature maps. A linear combination rule for individual features is most widely used [34], [35] where individual features are combined through a linear operation to form a final saliency map that guides attention. Top-down attention has been hypothesized to modulate the contribution from each map in an optimal manner [36] by adjusting biasing weights [37], [38]. Correlation between binary eye movements maps and feature similarity maps constructed by combining linearly the hue, frequency, and orientation similarity maps (appropriately weighted) should therefore be high.

We constructed similarity maps by linearly summing the individual feature similarity maps for all combinations of the three features, and found that the map formed from a linear combination of the hue and frequency maps (H+F), was most strongly correlated with eye movements.

To obtain an upper bound of correlation against which each rule in figure 7 could be compared, we created a maximum map (labeled “MaxMap” in the figure). The correlation values for this map were computed by taking the feature similarity map on each trial that had the strongest correlation with the saccade maps and storing this correlation value. The mean across trials was then computed from this trial-wise maximum, thus yielding an upper bound. We found that the map formed from the linear combination of hue and frequency (H+F map) was the closest to the upper bound. A significant effect of session on correlation values for this map was also observed (one-way ANOVA 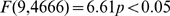). This suggests that subjects attended to the hue and frequency features and improved on the task by appropriately tuning top-down signals in the hue and frequency dimensions.

We also explored a multiplicative combination rule whereby we combined the maps in a point-wise multiplicative manner. Thus if a feature at a particular location is poorly matched to the target’s feature it will eliminate the chance for all other features to select this location as a potential target. This predicts a sparse saliency map, and has the elements of an AND operation on the multiple feature maps. However, if we look at the correlation values for the multiplicative map H*F*O they are not as strongly correlated as the H+F map. Despite the weak correlation we do find a trend in the correlation values for the H*F map (one-way ANOVA 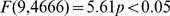). These results demonstrate a general improvement in the subjects’ tuning to the features of the target upon preview and also suggests that while the multiplicative rule makes for a computationally useful guidance strategy, a linear rule may be a more biologically plausible operation.

We then constructed a point-wise minimum map which would have the highest signal-to-noise ratio. The map was constructed by placing in each cell the value of the least similar item. In this manner the map contains low values in all locations except at the target cell location where the three feature maps would contain equal values. This strategy would call on a hypothetical observer to adopt the counter-intuitive strategy of searching for features that are most dissimilar to the target, thus highlighting a single location (target location) where no dissimilarities are found. However, it is difficult to conceive of a neural strategy that would enable such a mechanism since it would require pre-computation of all three feature maps, extraction of the most discriminative feature for each item, followed by construction of the final guidance map.

## Discussion

The triple conjunction search task learned by subjects in this study consisted of displays that remained consistent in the number of items and bottom-up uncertainty, however, the target changed both its location and features on each trial. Learning still took place under these conditions and the combined behavioral, occulomotor, and perceptual signatures of the improvement point towards effects beyond task acquisition. Behaviorally we saw an improvement in performance with subjects reporting the correct target on average 44% of the time at the beginning of the task to an average of 71% after developing expertise in this feature-rich environment. The occulomotor correlate of learning was evident from the changes in saccadic behavior, namely in the shorter ISI with training. Differences in basic saccade statistics in conjunction with visual search as well as learning have not been studied extensively. Phillips et al. [39] argue that gains in visual search performance are a result of an expansion in the ‘perceptual span’ and forward saccade amplitude, with a small effect of fixation duration which is equivalent to the ISI in our case. The improvement obtained in our case suggests both that there was an increase in perceptual span, as well as reduced dwell time for extracting information from each fixation.

Hooge & Erkelens [40] conducted experiments to specify the role of fixation duration in visual search tasks. The most salient feature of their study was the reconciliation of contradictory findings of [41] who found significant guidance of saccades towards items that were similar in color to the target, and Zelinsky [42] who did not find such guidance. Hooge & Erkelens [40] provide a means to make a leap from occulomotor dynamics to visual search performance using fixation duration as the vehicle for understanding the difference. They suggest that tasks involving difficult discriminations but easy peripheral selections tend to invoke longer fixation durations, while tasks involving easy discrimination but difficult peripheral selection (due to either an abundance or similarity of distractors around a target) tend to have shorter fixation durations but evoke a greater number of saccades. Our task is a difficult conjunction search where distractors share features with the target, this makes it a ‘hard-discrimination, hard-selection’ task. Therefore, initially we obtain high ISI’s (in fact ISI goes up from session one to session two) which perhaps suggests that our subjects’ occulomotor strategy focused on the foveal discrimination early in the task. High saccade count and reaction times suggest that the selection task was not easy either. However, with training we obtain much lower ISIs which implies that subjects improved on the discrimination task and could now concentrate resources on the selection task. Further, we find that the mean number of saccades stays fairly constant with subjects scanning over half the number of items on average. Thus, there is no significant change in the number of selections made during the search process, however, the ‘quality’ of the selections improves, i.e. the distractors chosen as potential targets are closer in their features to the target. The quicker ISIs may point toward an increased ‘perceptual span’ [43] or ‘visual lobe’ [44] that enables examination of a greater number of items in each saccade, however, additional experiments would be required to confirm this claim.

The occulomotor correlate of learning (i.e. improved discrimination by moving from discriminative search to selective search) then makes the prediction that subjects would have a higher tendency to make saccades towards patches that are similar to the target as they transition from discriminative search to selective search. Indeed this is what we found when we correlated saccade maps with feature similarity maps. By running a multiple logistic regression we found that whether a patch was selected for fixation could be predicted by the similarity of its features to the target and level of training of the subjects. These results on the similarity effect [45] serve as corroboration of several previous studies including [31] who found that monkeys make fixations to items that are similar in color but not orientation. Findlay & Gilchrist [45] also found a proximity effect, i.e., a tendency of saccades to fall near the target in space. Motter & Belky [31] also investigated this selection for color as a guiding feature over orientation. They conclude from their 1998 study, as well as electrophysiological studies in V4 [46], [47], that V4 neurons coded more strongly for stimuli in their receptive field that matched the top-down goal rather than the absolute color of the stimuli. This suggests that a color feature map would be the tool of choice for top-down attention in the guidance of saccades. Our study also demonstrates a preference for spatial frequency over orientation. Several other studies [32], [33] have found a similar preference for color as a guiding feature, and Wolfe & Horowitz [48] have placed color on top of the list of features that guide attention. We hypothesize that spatial frequency could be considered a ‘surface property’ much like texture and color that have desirous qualities for the guidance of attention. However, the current experiment does not address this feature-selective guidance and it would require further experiments to verify why orientation is a weaker cue for top-down attention in the presence of other features.

In this study the top-down goal changed on each trial and despite this we saw an increased similarity effect which suggests that activity of neurons in the visual cortex (e.g. V4 neurons) can be biased in a highly dynamic and rapid manner from one trial to the next. Therefore departing from typical perceptual learning studies we show evidence for learning that involves top-down processes. Herzog & Fahle [49] put forward a recurrent neural network model of perceptual learning that empahsizes the role of plasticity in the top-down connections as an enabling process for perceptual learning. They show that even in a task like vernier discrimination, where learning is both specific to stimulus features and spatial location, a model that incorporates top-down influences has more explanatory power than pure bottom-up models of improvement. Specifically they show that in a model where top-down connections gate flow of bottom-up inputs to decision units, learning acts upon the weights of the top-down connections rather than tuning properties of the bottom-up (sensory) inputs. The current study can also be placed in this context, situating the locus of plasticity in the top-down process rather than the bottom-up sensory process. However, in addition to this the increase in the similarity effect that we find, suggests that the ability to quickly switch the top-down signal also improved. It is certainly the case that there is a task-based effect and we cannot ascertain the exact amount of contribution which exclusive improvement in top-down biasing made toward progress in the task. However, it is clear from our analysis of correlation between feature similarity maps and binary saccade maps that there is enhanced guidance through better top-down biasing. We find that training enhances the similarity effect and a possible mechanism for this is improved top-down biasing. This enhances the right neurons which in turn guides attention to patches that are increasingly similar to the target.

Conjunction searches define targets using a combination of features, and binding of these features according to feature integration theory [34] requires attention. We examined the correlations of binary saccade maps and different combinations of feature similarity maps and found that a linear combination of the features hue and frequency was most highly correlated with saccade maps. We tried a multiplicative rule which provides the sparsest final similarity since it penalizes differences in a single feature while greatly boosting locations with a single matched feature. A similarity map constructed from a multiplication of hue and frequency was closely matched in terms of correlation with eye movements to the linear H+F map however, the H*F*O map was poorly corrleated with eye movements. A multiplicative rule however, does not account for the serial search times for conjunction searches since a precomputation of this multiplicative combination of features would put a hot-spot in a salience map at the location where all features match the target with high SNR. Overall this exploration points towards a linear combination rule that may be at play. That said, our discussion of the similarity effect also suggests a pre-attentive guidance of saccades towards potential targets. And if guidance is pre-attentive and feature combination requires attention, the prediction would be that conducting a conjunctive search is a serial process with respect to spatial attention and feature-based attention, and thus inefficient.
