# Correction: Training Top-Down Attention Improves Performance on a Triple-Conjunction Search Task

**DOI:** 10.1371/annotation/42360435-22f3-4185-a06b-3d7bbbd50352

**Published:** 2010-03-25

**Authors:** Farhan Baluch, Laurent Itti

An error in the author byline has been corrected. The new citation is: Baluch F, Itti L (2010) Training Top-Down Attention Improves Performance on a Triple-Conjunction Search Task. PLoS ONE 5(2): e9127. doi:10.1371/journal.pone.0009127.

